# miR-8 controls synapse structure by repression of the actin regulator Enabled

**DOI:** 10.1242/dev.105791

**Published:** 2014-05

**Authors:** Carlos M. Loya, Elizabeth M. McNeill, Hong Bao, Bing Zhang, David Van Vactor

**Affiliations:** 1 Department of Cell Biology and Program in Neuroscience, Harvard Medical School, Boston, MA 02115, USA; 2 Division of Biological Sciences, University of Missouri, Columbia, MO 65211, USA

**Keywords:** Enabled, NMJ, SSR, miRNA, Synapse, *Drosophila*

## Abstract

MicroRNAs (miRNAs) are post-transcriptional regulators of gene expression that play important roles in nervous system development and physiology. However, our understanding of the strategies by which miRNAs control synapse development is limited. We find that the highly conserved miRNA miR-8 regulates the morphology of presynaptic arbors at the *Drosophila* neuromuscular junction (NMJ) through a postsynaptic mechanism. Developmental analysis shows that miR-8 is required for presynaptic expansion that occurs in response to larval growth of the postsynaptic muscle targets. With an *in vivo* sensor, we confirm our hypothesis that the founding member of the conserved Ena/VASP (Enabled/Vasodilator Activated Protein) family is regulated by miR-8 through a conserved site in the Ena 3′ untranslated region (UTR). Synaptic marker analysis and localization studies suggest that Ena functions within the subsynaptic reticulum (SSR) surrounding presynaptic terminals. Transgenic lines that express forms of a conserved mammalian Ena ortholog further suggest that this localization and function of postsynaptic Ena/VASP family protein is dependent on conserved C-terminal domains known to mediate actin binding and assembly while antagonizing actin-capping proteins. Ultrastructural analysis demonstrates that miR-8 is required for SSR morphogenesis. As predicted by our model, we find that Ena is both sufficient and necessary to account for miR-8-mediated regulation of SSR architecture, consistent with its localization in this compartment. Finally, electrophysiological analysis shows that miR-8 is important for spontaneous neurotransmitter release frequency and quantal content. However, unlike the structural phenotypes, increased expression of Ena fails to mimic the functional defects observed in miR-8-null animals. Together, these findings suggest that miR-8 limits the expansion of presynaptic terminals during larval synapse development through regulation of postsynaptic actin assembly that is independent of changes in synapse physiology.

## INTRODUCTION

Synapses are the essential building blocks of neural circuitry. These highly specialized cellular junctions form and remodel under the control of various extrinsic cues and intrinsic regulatory mechanisms. Synaptogenesis is a coordinated process of cellular morphogenesis that requires localized and complementary construction of presynaptic and postsynaptic compartments (Collins and DiAntonio, 2007; Goda and Davis, 2003). Much attention has been focused on synaptic signaling pathways that promote the expansion of neuronal architecture in response to neuronal activity or extracellular cues. However, it is also clear that cell-intrinsic mechanisms are required to control the morphogenesis of synaptic structures. Indeed, intrinsic dysregulation of synaptic development can lead to a number of devastating neurological and psychiatric disorders (Arnsten et al., 2012). For example, cell-autonomous post-transcriptional regulation by the highly conserved Fragile-X Mental Retardation Protein (FMRP) is essential for limiting the expansion of both postsynaptic dendritic spines in mammals and presynaptic arbors in *Drosophila* (Gao, 2002).

Among the many intrinsic mechanisms that control synapse morphogenesis, there has been very rapid progress in our knowledge of post-transcriptional regulators. In particular, synaptic microRNAs (miRNAs) have emerged as a rich source of modulators for synapse form and function (McNeill and Van Vactor, 2012; Siegel et al., 2011). miRNAs are short ∼22 nucleotide (nt) non-coding RNAs known to control downstream gene expression by preferentially binding to complementary ‘seed’ sequences often located in the 3′UTR of target mRNAs (Bartel, 2009). miRNAs associate with target mRNA via protein complexes containing Argonaute (Ago)-family proteins that regulate mRNA stability and translation, thereby ‘tuning’ the level of protein produced by a target message (Bazzini et al., 2012; Guo et al., 2010). Recently, miRNAs have been shown to display developmentally and activity-regulated expression patterns in the brain and at the synapse (Kye et al., 2011; Lugli et al., 2008; Miska et al., 2004), suggesting underlying functions in nervous system development and synaptic plasticity. Although only a small fraction of these candidate miRNAs have been tested for synaptic functions *in vivo*, existing evidence implies that miRNA control of synapse form and function is quite complex (McNeill and Van Vactor, 2012; Mittelbrunn and Sánchez-Madrid, 2012). However, our understanding of the cell biological mechanisms by which specific miRNAs control synaptogenesis remains limited.

We have previously found that miR-8 is a potent regulator of neuromuscular junction (NMJ) morphogenesis in *Drosophila* (Loya et al., 2009). We also demonstrated that miR-8 controls presynaptic NMJ morphogenesis via tissue-specific activity in postsynaptic muscle cells. We identified the candidate effector protein Enabled (Ena), a founding member of the highly conserved Ena/VASP (Vasodilator-Stimulated Phosphoprotein) protein family. Endogenous levels of Ena are significantly increased in *miR-8*-null animals (Loya et al., 2009). Ena/VASP proteins are known to mediate multiple actin-based biological phenomena, such as axonal guidance, neurite initiation, neurotransmitter receptor packing, cell-cell junction maturation and cell motility (Drees and Gertler, 2008; Lin et al., 2010; Trichet et al., 2008; Vlachos et al., 2013). However, the morphogenetic contribution of Ena/VASP function at the synapse has not been defined in *Drosophila*.

In this study, we examined the developmental and cellular roles of miR-8 and Ena/VASP in postsynaptic muscle cells and investigated the functional relationship between these highly conserved genes. We find that miR-8 is required for NMJ expansion during the larval growth phase, and that Ena is a direct target of miR-8. Although Ena/VASP family proteins support both cell adhesive junctions and sites of actin-dependent membrane protrusion (Mittelbrunn and Sánchez-Madrid, 2012; Trichet et al., 2008), we find that the actin-assembly domains of an Ena ortholog are both sufficient and necessary to mediate both the synaptic localization and synapse growth-limiting functions of this conserved family of proteins. Together, these data support a model where postsynaptic modulation of Ena by miR-8 is part of an actin assembly-dependent cellular mechanism that coordinates presynaptic and postsynaptic morphogenesis during development.

## RESULTS

### miR-8 controls NMJ morphogenesis by limiting enabled levels

**Fig. 1.**
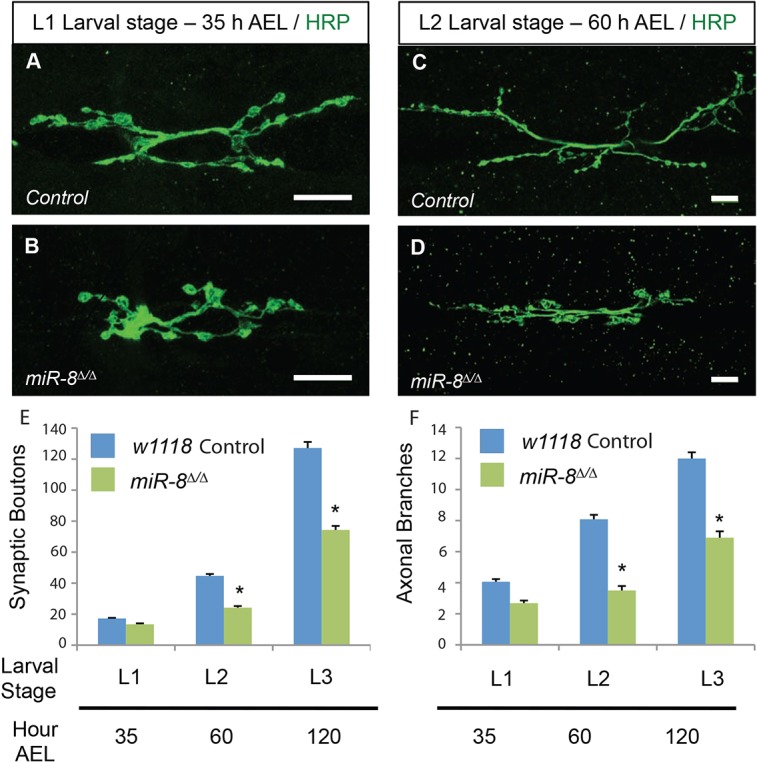
**miR-8 activity is predominantly required at L2 and L3 larval stages to promote neuromuscular junction development.** (A-D) Images of *w^1118^* (A,C) control and miR-8^Δ/Δ^ (B,D) *Drosophila* 6/7 NMJ at 35 h (mid-L1 stage; A,B) and 60 h (mid-L2 stage; C,D) after egg laying (AEL). Scale bars: 10 μm. Quantification of synaptic boutons (E) and axonal branches (F) shows a significant deficit in NMJ development at the L2 and L3 stages. Error bars indicate s.e.m. *P≤0.0001 relative to control animals (two-tailed Student's *t*-test). *n*≥12 for all genotypes.

Our prior end-point analysis showed that miR-8 is necessary for normal NMJ morphology at the end of the third instar (L3) stage (Loya et al., 2009). To determine the temporal onset of defects in synapse morphogenesis, we compared numbers of presynaptic boutons and branches at the well-characterized ventral NMJ (m6/m7) in wild-type and *miR-8*-null animals at larval instar stages L1, L2 and L3. We found that overall NMJ morphology appeared different from controls (Fig. 1A,B), but quantitative analysis of type 1b bouton and arbor branch addition did not reveal significant abnormality at L1 (Fig. 1E), whereas these parameters were consistently reduced in L2 and L3 compared with genetically matched controls (Fig. 1E). This developmental analysis prompted us to examine synaptic features that appear after L1, in the phase of NMJ expansion that is driven by rapid growth of the target muscles.

**Fig. 2.**
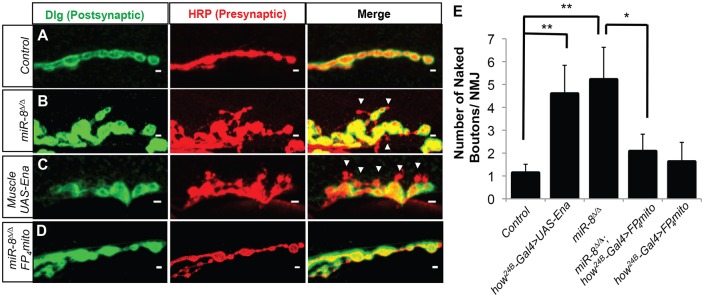
**miR-8 mutation disrupts synaptic specialization through regulation of Ena.** (A-E) Analysis of 6/7 NMJ boutons immunostained with postsynaptic marker Disc large (Dlg, green, left panels), presynaptic marker HRP (red, middle panels) and merged Dlg/HRP channel image (right panels). Control *w^1118^* (*n*=38) (A,E) display a low frequency of ‘naked’ synaptic boutons, as observed by the consistent apposition of HRP and Dlg signal. *miR-8*^Δ*/*Δ^ (*n*=38) (B,E) and muscle-specific overexpression of Ena (*UAS-Ena*) driven by *how^24B^-Gal4* (*n*=22) (C,E) NMJs causes significant (4.60- and 4.06-fold) increases, respectively, in ‘naked’ synaptic boutons. Expression of *UAS-FP4-mito* using the *how^24B^-Gal4* driver in a miR-8 homozygous mutant background (*n*=41) (D,E) significantly rescues the increase in naked bouton number in *miR-8*^Δ*/*Δ^ animals. Expression of *UAS-FP4-mito* using the *how^24B^-Gal4* driver (*n*=21) in a wild-type background shows no quantifiable difference from control. Scale bars: 2 μm. Error bars indicate s.e.m. **P≤0.01 relative to *w^1118^*, *P≤0.05 relative to *miR8*^Δ*/*Δ^ (two-tailed Student's *t*-test).

One feature of the NMJ that appears only after the L1 stage in type 1b synapses is the elaborate subsynaptic reticulum (SSR) of muscle membranes that houses postsynaptic cytomatrix components analogous to the junctional folds of vertebrate NMJs (Mosca and Schwarz, 2010; Rheuben et al., 1999; Sigrist et al., 2000). To evaluate NMJ structure, we used the synaptic scaffolding protein Discs large (Dlg; Dlg1 – FlyBase; Lahey et al., 1994), the *Drosophila* homolog of mammalian PSD-95, in combination with the *Drosophila* presynaptic membrane marker anti-horseradish peroxidase (α-HRP; Jan and Jan, 1982; see Materials and Methods). In wild-type m6/m7 NMJs, SSR-localized Dlg forms a halo around the HRP staining in type Ib boutons (Fig. 2A); boutons deficient in Dlg staining were rare in controls (approximately one per NMJ; Fig. 2E). However, in *miR-8* null animals, we found a fivefold increase in ‘naked’ boutons lacking a complete Dlg halo (Fig. 2B,E). These naked boutons were frequently small, suggesting a failure or delay in maturation.

Our previous studies suggested that postsynaptic-specific inhibition of Ena expression was sufficient to account for the presynaptic bouton growth, NMJ branching and arbor expansion activity of miR-8, as elevation of Ena in muscle, but not in neurons, mimics *miR-8* nulls (Loya et al., 2009). To determine whether elevation of Ena in muscle cells might produce a postsynaptic Dlg phenotype similar to that of *miR-8* mutants, we examined NMJs in animals where wild-type Ena cDNA under control of a Gal4 upstream activating sequence (UAS) was overexpressed with a muscle-specific Gal4 driver (how^24B^-Gal4). At the m6/m7 NMJs of these animals, we found naked boutons comparable to those observed in the *miR-8* null (Fig. 2C). The frequency of the naked boutons was also similar, suggesting that elevation of Ena is both qualitatively and quantitatively sufficient to account for the effect of the *miR-8*-null mutation (Fig. 2E). We next asked whether elevated Ena is necessary to induce the *miR-8*-null phenotype. Thus, we used a muscle-specific Ena dominant-negative (how^24B^-Gal4;UAS-mito-FP4; Gates et al., 2007), in combination with the *miR-8*-null mutant background, to prevent Ena elevation as a consequence of *miR-8* loss. The Ena dominant-negative rescued the *miR-8* mutant (Fig. 2D), reducing the number of naked boutons to background levels (Fig. 2E).

Our data suggest that Ena appears to be both a necessary and sufficient target downstream of postsynaptic miR-8; however, we needed to examine the mechanism by which miR-8 controls Ena expression. We first asked whether Ena is a direct target of miR-8 *in vivo.* We constructed a transgenic activity reporter in which the endogenous Ena 3′UTR was placed downstream of a Tubulin promoter-driven Enhanced Green Fluorescent protein (*EGFP-EnaUTR*) sequence (see Materials and Methods). Bioinformatic analysis showed that the Ena 3′UTR contains one highly conserved miR-8 seed sequence complement (TargetScanFly 6.2; Kheradpour et al., 2007). To determine whether this highly conserved site is important for regulation of the Ena 3′UTR by miR-8, we generated a control EGFP reporter where the seed sequence is mutated (*EGFP-EnaUTRmut*) (see Materials and Methods). We found that expression of UAS-miR-8 under the *ptc-Gal4* driver reduced the levels of EGFP-Ena3′UTR, but not of EGFP-Ena3′UTRmut, in the central region of the wing imaginal discs (supplementary material Fig. S1), as expected for a direct target gene. These findings suggest that miR-8 controls NMJ architecture by inhibiting directly the postsynaptic expression of Ena via a conserved target site in the 3′UTR of the Ena mRNA.

### Endogenous Enabled localizes in the postsynaptic peribouton area

**Fig. 3.**
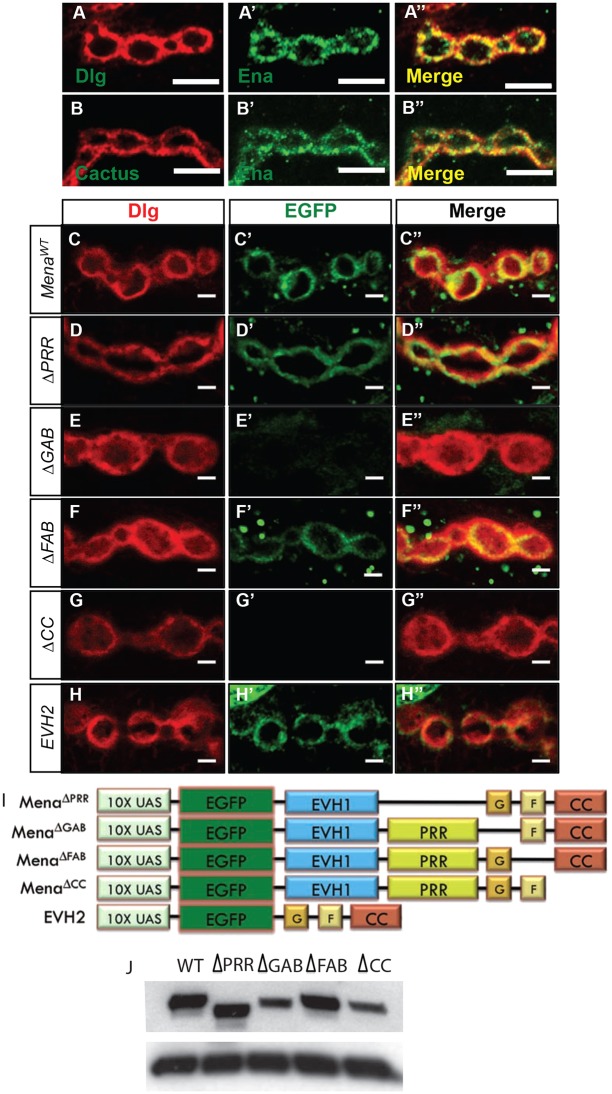
**Ena is enriched in SSR, and conserved actin-associated domains are necessary for synaptic localization.** (A-B) Images of synaptic boutons immunostained with postsynaptic markers Dlg (A) or Cactus (B), and Ena (A′,B′). Merged images of Ena/Dlg (A″) and Ena/Cactus (B″) show substantial colocalization (yellow). Scale bars: 5 μm. (C-H′′) Images of 6/7 NMJ boutons expressing wild-type or mutant *UAS-EGFP-mouse Ena* (*Mena*) transgenes using the *how^24B^-Gal4* driver. Immunostaining of Dlg (left panel), EGFP (middle panel) and Dlg/EGFP merge (right panel). Control *UAS-EGFP-Mena^WT^* boutons (C-C″) display colocalization between postsynaptic EGFP-Mena and Dlg, analogous to Ena immunostaining*. UAS-EGFP-Mena*^Δ*PRR*^ (D-D″) and *UAS-EGFP-Mena*^Δ*FAB*^ (E-E″) transgenes show EGFP-Mena staining pattern that is indistinguishable from wild-type control. Expression of *UAS-EGFP-Mena*^Δ*GAB*^ (F-F″) and *UAS-EGFP-Mena*^Δ*CC*^ (G-G″) demonstrate a marked deficiency in EGFP-Mena recruitment to the postsynaptic space. Expression of the N-terminal EVH2 domain (H-H″), which contains the GAB, FAB and CC motifs, shows localization that is indistinguishable from wild type at the synapse. Scale bars: 2 μm. (I) The UAS mammalian Ena (Mena) domain mutant transgenes (Loureiro et al., 2002) used to determine the structural requirements of Ena localization and function at the synapse. (J) Western blot analysis of *UAS-EGFP-Mena* transgenes driven by the *how^24B^-Gal4* driver show stable and comparable levels when probed with anti-EGFP (upper panel) and anti-tubulin as loading control (lower panel). Two whole animals were used per larval extract.

Ena is the founder of the conserved Ena/VASP family of proteins, which have well-established roles in formation of membrane protrusions and cell-cell adhesion (Drees and Gertler, 2008; Krause et al., 2003). Previous analysis revealed Ena accumulation at the NMJ (Martin et al., 2005). To determine whether Ena localization might account for a function within postsynaptic SSR, we performed confocal imaging of wild-type NMJs immunostained with both Ena and one of two well-characterized SSR markers: Dlg or Cactus (the *Drosophila* homolog of IκB) (Heckscher et al., 2007). Although we observed some clusters of Ena protein within the presynaptic terminal and in punctate structures within the muscle surrounding the SSR, Ena was most highly enriched in the peribouton area surrounding the presynaptic compartment (Fig. 3A′,B′). Ena colocalized significantly with postsynaptic Dlg and Cactus proteins, but was frequently concentrated in a region most proximal to the synaptic membrane contact between boutons and SSR when compared with Dlg, suggesting preferential association with the postsynaptic membrane (see merged channels in Fig. 3A″,B″).

### Conserved domains are necessary and sufficient for Mena postsynaptic localization

Recent studies have revealed a vital role for the postsynaptic actin cytoskeleton in the regulation of synapse shape, growth and function (Okamoto et al., 2004; Coyle et al., 2004). Ena/VASP family proteins mediate actin assembly via a set of highly conserved C-terminal domains (Ahern-Djamali et al., 1998). In order to determine whether conserved Ena family actin-regulatory domains were necessary or sufficient for localization in the SSR, we generated a series of transgenic animals harboring an inducible UAS-EGFP-mammalian Ena (Mena) fusion protein with one of several mutations in the conserved C-terminal domains known to be important for actin assembly (supplementary material Fig. S2A). We used *Mena* transgenes because: (1) Mena structural mutant constructs have been previously characterized in detail (Loureiro et al., 2002); (2) there is a high degree of sequence and functional conservation, underscored by the rescue of *Drosophila ena* mutant lethality by mammalian orthologs (Ahern-Djamali et al., 1998); and (3) Mena transgenes are resistant to RNA interference (RNAi)-mediated knock down of endogenous *Drosophila ena*.

The highly conserved structure of Ena/VASP consists of an N-terminal Ena/VASP Homology 1 (EVH-1) domain, a central proline-rich region (PRR) that is known to bind Profilin and to other SH3-containing proteins, and a C-terminal Ena/VASP Homology 2 (EVH2) domain (Bear and Gertler, 2009) (supplementary material Fig. S2A). Notably, the conserved EVH2 domain, sufficient to mediate Ena/VASP actin assembly, is composed of: (1) a Thymosin β4-like motif that is related to the globular actin-binding (GAB) site of Thymosin β4 (Van Troys et al., 1996); (2) an F-actin binding (FAB) motif (Lambrechts et al., 2000); and (3) a predicted coiled-coil (CC) region that can mediate Ena oligomerization (Ahern-Djamali et al., 1998; Carl et al., 1999). To examine the protein product of the EGFP-Mena structural mutants, we performed western blot analysis of whole-larva extracts of each *how^24B^-Gal4:UAS-EGFP-Mena* transgene. We confirmed using immunoblot analysis that EGFP-Mena mutant proteins are expressed at levels comparable to wild-type EGFP-Mena, albeit slightly lower for GAB and CC mutants, and they migrate to their predicted size (supplementary material Fig. S2B).

To assess Mena localization in the postsynaptic space, we expressed wild-type Mena (*UAS-EGFP-Mena^WT^*) and structural mutant transgenes using the pan-mesodermal *how^24B^-Gal4* driver, and immunostained with anti-EGFP and anti-Dlg antibodies. Analysis of *UAS-EGFP-Mena^WT^* revealed peribouton area recruitment and colocalization with the bouton-proximal domain of endogenous Dlg (Fig. 3C-C″) indistinguishable from endogenous Ena immunostaining. Deletion of the conserved proline-rich region (*UAS-EGFP-Mena*^Δ*PRR*^), as well as mutation of the F-actin binding domain (*UAS-EGFP-Mena*^Δ*FAB*^) showed wild-type SSR localization (Fig. 3D-D″,F-F″). These findings suggest that in the presence of endogenous wild-type Ena, the proline-rich region and the F-actin binding domain are not structurally required for Mena recruitment to the postsynaptic terminal. Conversely, expression of *UAS-EGFP-Mena*^Δ*GAB*^ or *UAS-EGFP-Mena*^Δ*CC*^ transgenes, which contain deletions in the G-actin binding or coiled-coil motifs, respectively, revealed a striking deficit in the recruitment of EGFP-Mena to the peribouton area (Fig. 3E-E″,G-G″). As these regions of the EVH2 domain are vital for Ena/VASP-dependent actin assembly (Bear and Gertler, 2009), we asked whether the C-terminal EVH2 domain containing all of the essential actin assembly function is sufficient to localize Mena in the SSR. Indeed, expression of the EVH2 domain alone (*UAS-EGFP-EVH2*), which contains the GAB, FAB and CC motifs, showed recruitment to the peribouton space indistinguishable from wild-type Ena and Mena proteins (Fig. 3H-H″).

### Postsynaptic Ena/VASP function is actin dependent

**Fig. 4.**
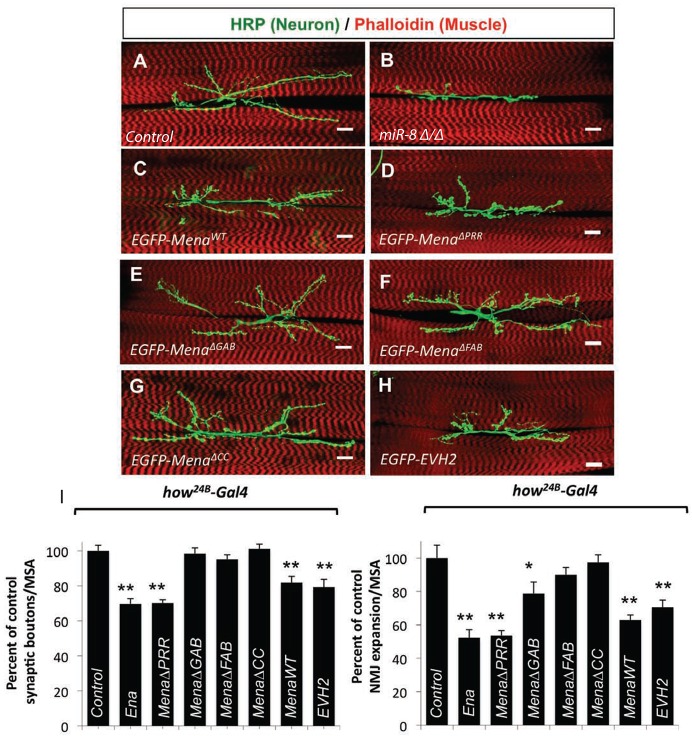
**Conserved actin-associated domains are required for Mena function at the synapse.** (A-H) Images of *Drosophila* 6/7 NMJ expressing wild-type or mutant *UAS-EGFP-Mena* transgenes using the pan-muscle *how^24B^-Gal4* driver. Scale bars: 20 μm. Muscle expression of *UAS-EGFP-Mena^WT^* (C), *UAS-EGFP-Mena*^Δ*PRR*^ (D) and *UAS-EGFP-EVH2* (H) show significantly disrupted NMJ morphogenesis. (I) Quantification of synaptic boutons and NMJ expansion. Expression of *UAS-EGFP-Mena*^Δ*GAB*^ (E), *UAS-EGFP-Mena*^Δ*FAB*^ (F) and *UAS-EGFP-Mena*^Δ*CC*^ (G) is indistinguishable from wild-type control NMJs. Error bars indicate s.e.m. *P≤0.01, **P≤0.001 relative to *how^24B^-Gal4/+* animals (two-tailed Student's *t*-test). *n*≥17 for all genotypes and parameters.

Elevation of postsynaptic Ena during NMJ development restricts presynaptic arbor expansion comparable to loss of the Ena inhibitor miR-8 (Loya et al., 2009). To determine which conserved C-terminal domains are required for this activity of Ena/VASP proteins, we quantified NMJ morphometry in animals expressing each of the UAS-EGFP-Mena transgenes with *how^24B^-Gal4*. Overexpression of wild-type *Mena* reduced synaptic bouton number and arbor area compared with controls (Fig. 4D,I), and were comparable in significance to overexpression of Ena (Fig. 4C,I). When we assayed the panel of *Mena* deletion mutants, however, only *UAS-EGFP-Mena*^Δ*PRR*^ was equally effective in limiting NMJ growth when compared with the wild-type Ena and Mena transgenes (Fig. 4E,I). The remaining mutants all failed to limit NMJ growth when compared with Gal4 controls (Fig. 4E-G,I). Thus, both of the actin-binding domains and the C-terminal coiled-coil domain are required for the NMJ-limiting activity of Mena. As all of the conserved components of EVH2 were necessary for Mena function, we asked whether EVH2 alone might be sufficient to limit NMJ growth. Indeed, expressing *UAS-EGFP-Mena^EVH2^* produced the same effect as expressing wild-type Mena under the same conditions (Fig. 4H,I). Because elevation of postsynaptic EVH2 domain function was both necessary and sufficient to mimic elevation of Ena, and to localize Mena in the SSR, we hypothesized that Ena/VASP-dependent actin assembly is responsible for limiting presynaptic morphogenesis during larval development.

**Fig. 5.**
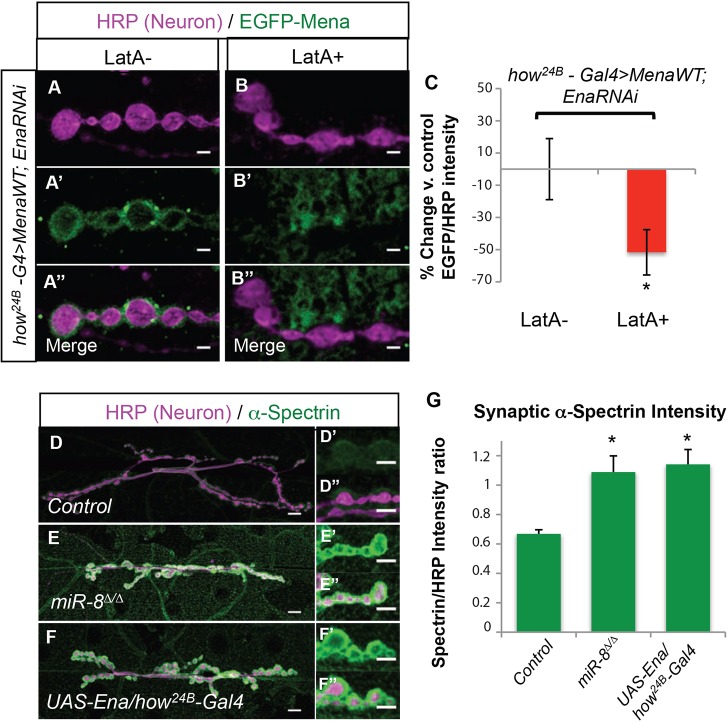
**Disruption of actin polymers reduces synaptic localization of Mena, and Ena can increase actin-associated protein abundance postsynaptically.** (A-B″) Images of synaptic boutons immunostained with HRP and EGFP. Scale bars: 2 μm. Muscle expression of *UAS-EGFP-Mena^WT^* and *UAS-EnaRNAi* show peri-synaptic bouton localization similar to animals expressing endogenous Ena in the background (A-A″). *EGFP-Mena^WT^* localization is disrupted by LatA treatment (B-B″). Quantitative immunohistochemical (QIHC) analysis shows a significant decrease in *EGFP-Mena^WT^* intensity in the peri-synaptic bouton area (C). (D-F″) Images of the 6/7 NMJ immunostained for HRP and α-Spectrin: *w^1118^* control (D-D″), *miR-8*^Δ*/*Δ^ (E-E″) and *UAS-Ena/how^24B^-G4*. Scale bars: 10 μm (D,E,F) and 5 μm (D′,D″,E′,E″,F′,F″). Quantitative immunohistochemistry shows a significant increase in α-Spectrin levels in *miR-8*^Δ*/*Δ^ and *UAS-Ena/how^24B^-Gal4* NMJs (G). Error bars indicate s.e.m. *P≤0.05, relative to control animals (two-tailed Student's *t*-test). *n*≥9 for all genotypes.

The role of EVH2 in the localization and function of postsynaptic Ena/VASP suggested that SSR morphogenesis and control of presynaptic growth depends on postsynaptic actin assembly. However, Ena/VASP localization to regions of actin-dependent membrane protrusion also requires F-actin itself (Trichet et al., 2008). To provide a further test of our model, and to confirm that EGFP-Mena localizes to the SSR in the absence of endogenous Ena, we expressed in muscle *UAS-EGFP-Mena^WT^* in the presence of *UAS-Ena^RNAi^*. Even when muscle Ena is knocked down to low levels, wild-type Mena localizes to SSR (Fig. 5A-A″). We then prepared live larval fillets in physiological solution and then bathed the NMJ in the actin depolymerizing drug Latrunculin A (LAT A) and compared the results with vehicle-only controls (see Materials and Methods). After a brief exposure (10 min) to Lat A, postsynaptic localization of Mena was significantly reduced when compared with vehicle controls using quantification of fluorescence intensity (Fig. 5A-C), thus confirming the actin dependence of synaptic Ena/VASP localization, analogous to Ena/VASP localization at the tips of growth cone filopodia (Lanier and Gertler, 2000). Staining of these pelts with anti-Dlg revealed no obvious difference between control and Lat A-treated samples (data not shown).

If Ena/VASP-dependent actin assembly in the SSR accounts for the growth limiting effects of the *miR-8*-null mutant, we would further predict that elevation of Ena and loss of miR-8 would both induce an increase in periboutonal actin polymer content. Although distinguishing F-actin staining at synaptic sites embedded beneath the surface of an actin-rich muscle cell is extremely difficult, other groups have exploited actin-associated proteins as markers for the distribution of F-actin in different contexts (Ramachandran et al., 2009; Ruiz-Cañada et al., 2004). We incubated NMJs from *miR-8* mutants and muscle-specific Ena overexpression genotypes with antibodies to α-Spectrin, the localization of which is dependent on F-actin (Ruiz-Cañada and Budnik, 2006), and stained using secondary antibodies. Quantification of α-Spectrin levels in confocal optical sections shows comparable and significant increases in peribouton localization in both of these genotypes compared with controls (Fig. 5D-G).

### Actin-capping protein antagonizes postsynaptic Ena function

**Fig. 6.**
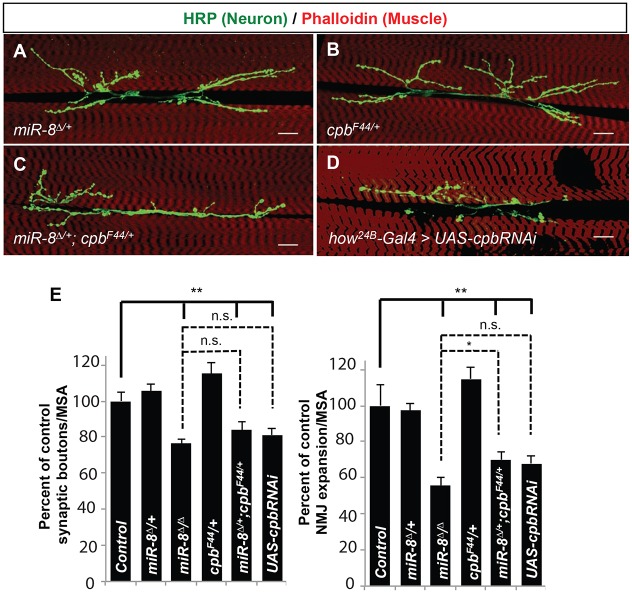
**Capping protein β genetically interacts with miR-8 to antagonize Ena activity at the synapse.** (A-D) Images of the 6/7 NMJ showing *miR-8*^Δ*/+*^ (A), *cpb^F44/+^* (B), *miR-8*^Δ*/+*^*; cpb^F44/+^*(C) and *how^24B^-Gal4/UAS-cpbRNAi* (D). Scale bars: 20 μm. (E) Quantification of synaptic boutons and NMJ expansion. Error bars indicate s.e.m. *P≤0.05, **P≤0.001, relative to control animals (two-tailed Student's *t*-test). *n*≥12 for all genotypes.

In addition to recruiting G-actin monomers and Profilin-Actin complexes to the barbed ends of membrane-proximal microfilaments, Ena/VASP association with the barbed end also inhibits the binding of polymer-terminating actin-capping proteins (Gates et al., 2009; Bear and Gertler, 2009). This provided an additional testable prediction: actin-capping protein β (Cpb) should act as an antagonist of Ena and cooperate with miR-8 to promote NMJ growth. We first tested for cooperation between miR-8 and Cpb using a well-established trans-heterozygous complementation assay for dose-dependent genetic interaction. Although animals heterozygous for nulls of either *miR-8* (*miR-8***^Δ/^***^+^*) or *cpb* (*cpb^F44/+^*) alone displayed normal NMJ morphology (Fig. 6A,B,E), the trans-heterozygotes (*cpb^F44/+^, miR-8***^Δ*/*^***^+^*) showed decreases in presynaptic boutons and NMJ area similar to *miR-8* homozygotes (Fig. 6C,E). To provide an independent test that Cbp is required in muscle to restrict NMJ growth, we deployed *cpb-RNAi* using muscle-specific Gal4. As predicted by our model, postsynaptic knockdown of Cbp caused reductions in presynaptic bouton number and NMJ area that were qualitatively and quantitatively indistinguishable from loss of *miR-8* (Fig. 6D,E). These data support a model where Ena restricts NMJ growth via barbed-end actin assembly within the SSR, but also suggest that normal NMJ growth depends on postsynaptic actin remodeling under a regulatory balance of capping and anti-capping activities.

### miR-8 regulation of postsynaptic ultrastructure is Ena-dependent

**Fig. 7.**
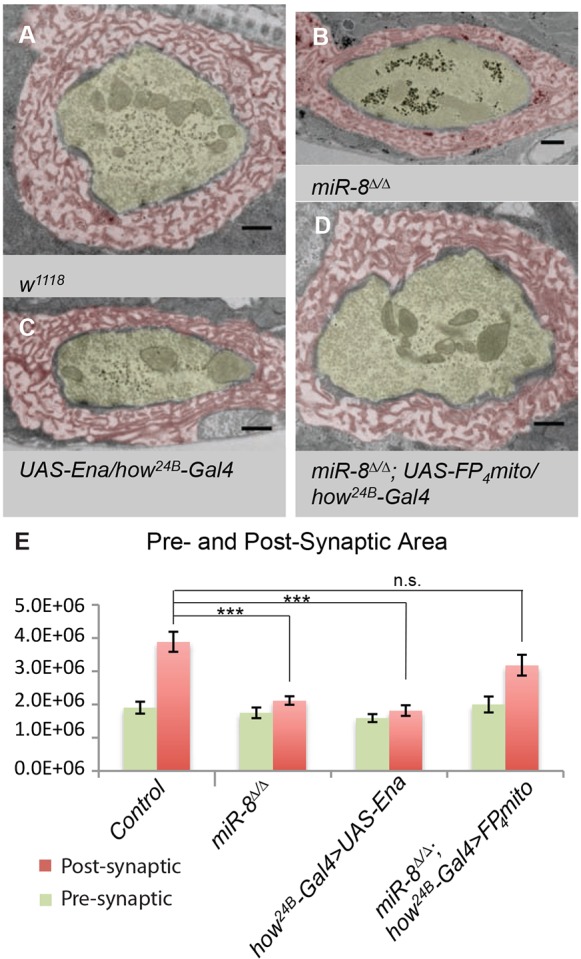
**miR-8 mediates SSR elaboration through repression of Ena activity.** (A-E) Analysis of electron micrographs of type Ib synaptic boutons at the 6/7 NMJ. (A-D) SSR (pseudocolored red) and presynaptic bouton (pseudocolored yellow). Representative image of a control *w^1118^* (A) synaptic bouton with surrounding SSR. Both *miR-8*^Δ*/*Δ^ (B) and *how^24B^-Gal4>UAS-Ena* (C) display significant reduction in SSR area relative to control (A). Expression of *how^24B^-Gal4>UAS-FP_4_-mito* in a *miR-8*^Δ*/*Δ^ background significantly rescues all synaptic bouton elaboration defects observed in miR-8 homozygous mutants presynaptically (green) and postsynaptically (red, D). Scale bars: 500 nm. SSR thickness and area of *w^1118^* (*n*=23), *miR-8*^Δ*/*Δ^ (*n*=31), *how^24B^-Gal4>UAS-Ena* (*n*=23) and *miR-8*^Δ*/*Δ^*;* *how^24B^-Gal4>UAS-FP_4_-mito* (*n*=21). Error bars indicate s.e.m. ***P≤0.0001, **P≤0.001, relative to *w^1118^* control (two-tailed Student's *t*-test).

Our analysis of synaptic phenotypes and protein localization strongly suggested that miR-8 regulates NMJ architecture through Ena function within the SSR; however, our assessment of SSR structure was based on light level markers. The most accurate high-resolution method to assess SSR architecture remains transmission electron microscopy (TEM). When we compared *miR-8*-null NMJ ultrastructure with that of genetically matched controls (*w^1118^*), we found overall SSR to be substantially diminished (Fig. 7A,B). Quantitative analysis of electron micrographs from *miR-8*-null mutant m6/m7 NMJs revealed a significant reduction in SSR thickness (37%, *P*<10^−9^) (Fig. 7E), area (45%, *P*<10^−6^) and fold complexity (30%, *P*<0.01, see Materials and Methods) relative to control. Measurement of presynaptic bouton area revealed no significant alteration in miR-8 mutants (Fig. 7E). The same was true of other presynaptic features [active zone (AZ) length and number of T-bars per AZ; Table 1], consistent with light level analysis of presynaptic active zone markers such as Bruchpilot (Brp; not shown). Thus, TEM showed that miR-8 is necessary to promote and/or maintain normal postsynaptic SSR morphogenesis.

**Table 1.**
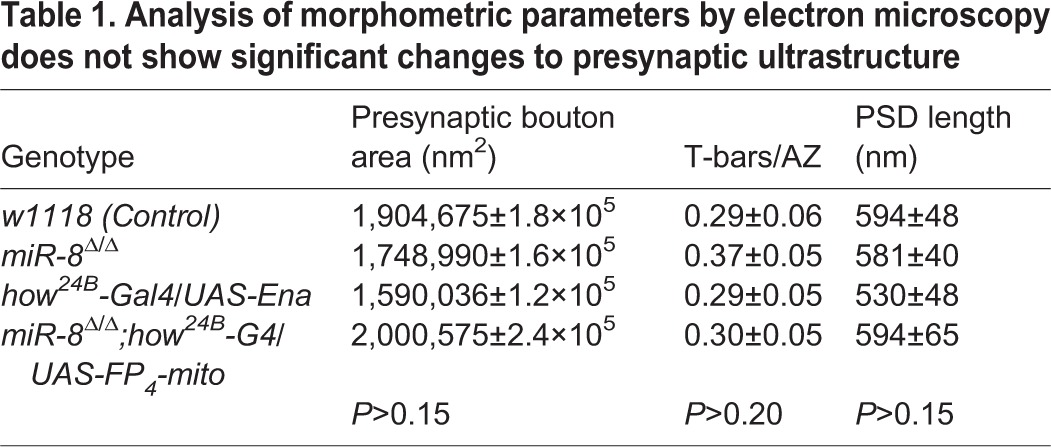
**Analysis of morphometric parameters by electron microscopy does not show significant changes to presynaptic ultrastructure**

To determine whether postsynaptic elevation of Ena alone might recapitulate the SSR ultrastructure defects that we found in *miR-8* mutants, we used TEM to analyze animals where *UAS-Ena* was expressed in muscle using *how^24B^-Gal4*, and found a phenotype that was both qualitatively and quantitatively similar to loss of miR-8 (Fig. 7C,E). We observed that expression of *UAS-Ena* results in a significant decrease in SSR thickness (46%, *P*<10^−9^), area (53%, *P*<10^−6^) and fold complexity (35%, *P*<0.02), similar to *miR-8*-null mutant synapses. Similar to the miR-8 phenotype, we found no change in presynaptic area with Ena overexpression in muscle (Fig. 7E). To complement the gain of function, when the Ena dominant-negative was combined with a *miR-8*-null mutant background, the SSR ultrastructure defect was rescued (Fig. 7D). Quantitative analysis confirmed that SSR area in the double mutant was no longer significantly different from controls (Fig. 7E). Thus, inhibition of Ena expression in muscle was sufficient to account for the observed abnormalities in postsynaptic architecture, caused by loss of miR-8.

### miR-8 regulates synaptic transmission independently of postsynaptic Ena

**Fig. 8.**
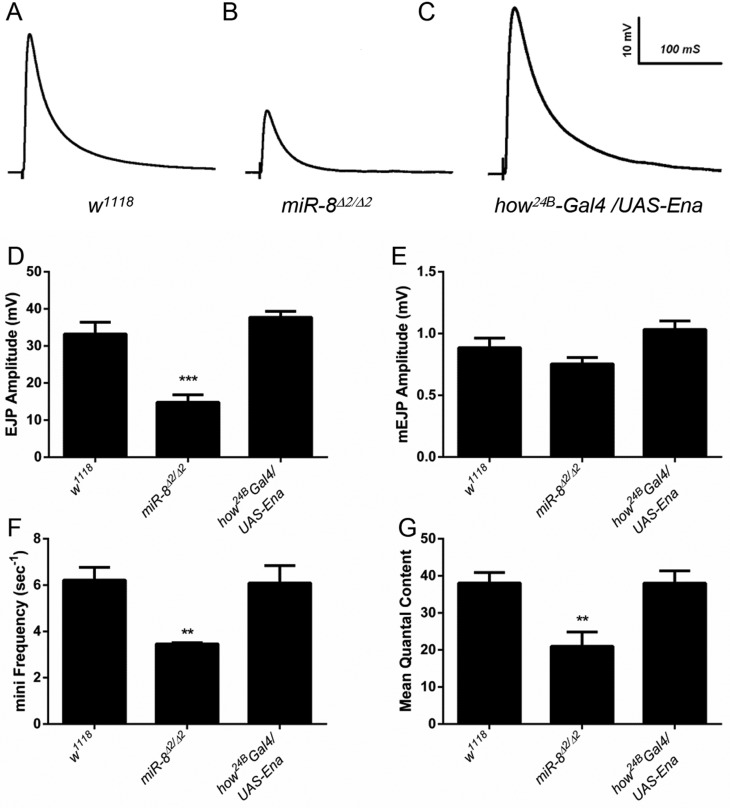
**Abnormal synaptic transmission of *miR-8* mutant NMJs.** (A-C) Representative traces of evoked excitatory junction potentials (EJPs) recorded from muscle 6 of the NMJ: (A) *w^1118^*, (B) *miR-8^Δ2/Δ2^* and (C) *how^24B^-Gal4/UAS-Ena.* (D-G) Quantitative histograms show a significant reduction in evoked EJP amplitude (mV), mini frequency (s^−1^) and mean quantal content of *miR-8^Δ2/Δ2^*, but not *how^24B^-Gal4/UAS-Ena*, relative to *w^1118^* control. ***P<10^−4^, **P<0.003 (two-tailed Student's *t*-test).

The striking effects of *miR-8* loss on synaptic morphology raised the issue of whether these defects in NMJ expansion and SSR ultrastructure might be associated with altered synaptic transmission. Therefore, we recorded and compared evoked excitatory junctional potentials (EJP) in wild-type and *miR-8*-null animals, and found a highly significant decrease in average EJP amplitude (Fig. 8A,B,D). However, a reduced EJP could result from pre- and/or postsynaptic defects, so we also compared the amplitude and frequency of spontaneous release. Interestingly, we found that the decrease in amplitude of miniature potentials was not significant, suggesting that postsynaptic responses were relatively normal (Fig. 8E). The frequency of miniature potentials, however, showed a highly significant halving in miR-8 nulls compared with control (Fig. 8F), indicating a presynaptic defect in neurotransmitter release and mean quantal content (Fig. 8G). We reasoned that such a defect in presynaptic glutamate release could reflect some trans-synaptic effect of Ena regulation by miR-8 in muscle, as we found for bouton growth and branching; or the decreased release could reflect presynaptic regulation of some unidentified target gene in motoneurons. Indeed, although our previous results showed presynaptic Ena to be refractory to miR-8 inhibition, we did find modest neuronal activity of miR-8 with a synthetic activity sensor (Loya et al., 2009). Thus, we recorded EJP and miniature events in animals overexpressing Ena in muscle (using how^24B^-Gal4). To our surprise, despite clear NMJ morphology phenotypes in these Ena overexpressors, there were no significant changes in synaptic transmission (Fig. 8A,C-G).

## DISCUSSION

A number of intrinsic cellular mechanisms control the morphogenesis of synapses during their initial formation, developmental remodeling and activity-dependent plasticity. Among the post-transcriptional regulators of synaptogenesis, relatively few miRNA-dependent mechanisms have been examined in detail. Our current study demonstrates that the highly conserved actin-regulatory protein Ena is a bone fide target of miR-8. Detailed analysis of miR-8 and Ena function at the larval NMJ reveal that miR-8 promotes the expansion of presynaptic arbors and the elaboration of a complex postsynaptic membrane structure that surrounds these presynaptic terminals via inhibition of Ena. Postsynaptic Ena/VASP proteins localize to the very same bouton-proximal region of the subsynaptic reticulum (SSR) that requires miR-8. Loss and gain of function for *Drosophila* Ena demonstrates that Ena is both sufficient and necessary for this postsynaptic output of miR-8. Structure-function experiments suggest that both the localization and NMJ growth-limiting function of Mena requires conserved C-terminal actin-regulatory domains. Moreover, Mena NMJ localization is dependent upon F-actin, as has been previously observed for Ena/VASP proteins in regions of leading-edge membrane protrusion in motile cells and growth cones (Trichet et al., 2008). Our findings suggest a model where miR-8 promotes NMJ growth by limiting levels of postsynaptic Ena-dependent actin assembly. In accordance with this model, we find that actin capping protein (Cpb) cooperates with miR-8 to regulate NMJ expansion.

In *Drosophila*, as in vertebrates, NMJs are embedded in the muscle surface, surrounded by complex infolded muscle membranes rich in postsynaptic cytomatrix and neurotransmitter receptors. The intricate SSR architecture at *Drosophila* NMJs is constructed later than initial presynaptic bouton morphogenesis (Budnik, 1996). Because new boutons are added to expand the arbor in the L2 and L3 instars by a budding process from interstitial or terminal boutons, late NMJ growth must involve remodeling of the SSR in order to accommodate nascent boutons (Mosca and Schwarz, 2010). Our analysis suggests that enhanced postsynaptic actin assembly within the SSR limits presynaptic growth. A simple model to explain this effect is that the protrusion of presynaptic membrane during bouton initiation requires a coordinated reduction in the actin-dependent protrusion of opposing postsynaptic membranes. Such a model can explain why loss of postsynaptic actin capping (Cpb) and elevation of anti-capping (Ena) effectors have equivalent effects on presynaptic morphogenesis. This model also predicts that stable synapses may be subject to a balanced reciprocal force produced by continued actin polymer turnover.

Comparison of naked bouton structure in *miR-8* mutant and Ena overexpression NMJs shows a substantial resemblance to ‘satellite’ boutons, small ectopic boutons emanating from axonal branches, previously reported in studies where pathways of cell-adhesion (Fas2), endocytosis (Endophilin) and presynaptic actin dynamics (Cyfip; Sra-1 – FlyBase) were disrupted (Torroja et al., 1999; Ashley et al., 2005; Dickman et al., 2006; Zhao et al., 2013). Although similar in morphology, the underlying postsynaptic molecular architecture described in these previous studies appears more intact, as evidenced by the colocalization of postsynaptic marker Dlg (Torroja et al., 1999; Ashley et al., 2005; Zhao et al., 2013). Although the importance of Dlg at the synapse to satellite budding and stability is unknown, it may be useful for understanding the interplay of miR-8 with Fas2, Endophilin and/or Cyfip in regulation of synapse development. Because multiple signaling pathways are known to control NMJ morphogenesis (Johnson et al., 2006; Keshishian and Kim, 2004; Korkut et al., 2009), it is tempting to speculate that the postsynaptic miR-8/Ena mechanism may be coupled to upstream signals or to synaptic activity in order to create conditions permissive to NMJ expansion. Future studies will be required to define factors upstream of miR-8 activity in muscle.

It has been proposed that SSR houses machinery for local protein synthesis of postsynaptic glutamate receptors (Sigrist et al., 2000), raising the issue of whether reductions in SSR that are induced by loss of miR-8 might have an indirect impact on NMJ physiology. The reduction in the frequency of spontaneous release and in quantal content observed in *miR-8* nulls but not in Ena overexpressors further implies that miR-8 has yet additional target genes in the presynaptic compartment. Therefore, we believe that the role of miR-8 in controlling SSR, bouton and branch formation relates primarily to the coordinated morphogenesis required for NMJ expansion in later larval life. In conclusion, our studies uncover the novel miR-8/Ena regulatory axis of NMJ morphogenesis that involves the modulation of the underlying postsynaptic actin cytoskeleton, thereby expanding our knowledge of the diverse repertoire of microRNA regulation in synapse development.

## MATERIALS AND METHODS

### *Drosophila* genetics

All stocks were maintained at 25°C according to standard procedures. Stocks were obtained from the Bloomington Stock Center (Bloomington, IN, USA) unless otherwise specified. The following Gal4 drivers were used: *ptc-Gal4*, *elav-Gal4* and *how^24B^-Gal4*. The *miR-8*^Δ*/*Δ^ stock described in this study was generated in the Van Vactor laboratory (Loya et al., 2009). *UAS-EnaRNAi* and *UAS-cpbRNAi* was obtained from Vienna Drosophila RNAi Center (VDRC, Vienna, Austria), *cpb^F44^* was a gift from P. Garrity (Waltham, MA, USA), *miR-8*^Δ*2/*Δ*2*^ was a gift from S. Cohen (Proteos, Singapore), and *UAS-FP_4_-mitoEGFP* was a gift from M. Peifer (Chapel Hill, NC, USA). The specificity of the *UAS-FP_4_-mito* has been previously described (Gates et al., 2007).

### Molecular biology

Generation of the EGFP-Ena-3′UTR sensor transgene involved amplification of the endogenous Ena 3′UTR sequence from *Drosophila* genomic DNA [PCR primer sequences: F, 5′ aaaaaaGCGGCCGCA- TCAAAATGCTGTCACGATAAACGCGA3′; R, 5′aaaaaaTCTAGATGT-TTCTGATTTGCTGAAGACTTGCTGG3′; EGFP-Ena sequence (uppercase), 5′ end sequence (lowercase) was added to ensure restriction enzyme cleavage], cloning into pUAST-EGFP vector using *Not*I and *Xba*I restriction endonuclease enzymes (NEB). For EGFP-Ena-3′UTR mutant sensor, the conserved miR-8 target site was mutated from TAGTATTA to CCTGGCG (mutagenesis primer sequences: F, 5′GGACTACTCATTAAACTAACCTAAAGGGAACTcctggcg-CGACTCAAAAACGAAATGAAAACAATTCC3′; R, 5′GTTTTCATTTC-GTTTTTGAGTCGcgccaggAGTTCCCTTTAGGTTAGTTTAATG3′) using the Stratagene QuikChange Site-Directed Mutagenesis Kit (Santa Clara, CA, USA). Transgenic animals were generated following P element transposon-mediated genomic integration (Genetic Services, Cambridge, MA, USA).

Mena structural mutant vectors were a gift from F. Gertler (Loureiro et al., 2002). We amplified the EGFP-Mena transgenes in wild-type form, EVH2 domain only, or harboring deletion in the conserved proline-rich region (PRR), G-actin binding (GAB), F-actin binding (FAB) or coiled-coil (CC) motifs by PCR (PCR primer sequences: F, 5′AAAAAGAATTCCAAAA-TGGTGAGCAAGGGCGAGGAGCTGTTCACC3′; R, 5′AAAAATCT-AGAAAAAGCTAGCTTGCCAAACCTACAGGTGGGGTCT3′). To avoid variability in expression between the different EGFP-Mena transgenes, we cloned each PCR fragment into the pWalium10 (a gift from N. Perrimon; Ni et al., 2008) vector using the *Eco*RI and *Nhe*I restriction endonuclease enzymes (NEB). Transgenic animals were generated following phiC31-targeted integration method (57) into attP2 docking sites located in the third chromosome (Genetic Services, Cambridge, MA, USA).

### Immunohistochemistry and quantitation of NMJ development

Wandering third instars were dissected in Ca^2+^-free saline and fixed in 4% paraformaldehyde for 30 min, except for Ena immunostaining, which required a fixation in a mix of 37% formaldehyde and 100% methanol for 10 min. The following primary antibodies were used for immunohistochemistry: anti-HRP, 1:1000 (123-095-021; Jackson ImmunoResearch, West Grove, PA, USA); FITC-conjugated anti-GFP, 1:1500 (ab6662; Abcam, Cambridge, MA, USA); and anti-Ena 1G6, 1:4 (a gift from F. M. Hoffmann, Madison, WI, USA). Antibodies obtained from the Developmental Studies Hybridoma Bank Iowa City, IA, USA include: anti-α-Spectrin 3A9, 1:30; anti-Cactus 3H12, 1:100; and anti-Dlg 4F3, 1:100. F-actin was visualized using Alexa Fluor 633 phalloidin (1:400; Invitrogen). Secondary antibodies conjugated with fluorophores FITC, Cy3 and AMCA (Jackson ImmunoResearch, West Grove, PA, USA) were used at a 1:200 dilution. RP3 and MN6/7b terminals of muscle 6 and 7 in the abdominal segment A2 of wandering third instar larvae were used for the quantification of all morphological parameters. This analysis was carried out using a Zeiss Axioplan2 microscope and a Hamamatsu ORCA wide-field digital camera as previously described (Loya et al., 2009). Bouton quantification was performed as previously described (Mosca and Schwarz, 2010).

### Confocal and epifluorescence microscopy

Confocal microscopy was performed using a Zeiss LSM 510 META upright microscope. Max-intensity projections were obtained using the Zeiss LSM Software package. For gross NMJ morphology, stacks of A2 6/7 NMJs were obtained under identical conditions. Prior to acquisition, laser parameters were adjusted to obtain non-saturating conditions. Fluorescent signal intensity was quantified using an ImageJ macro to quantify intensity relative to HRP in an area approximately 0.5-1 µm around the HRP staining. Images for mutant transgenes EGFP-Mena^ΔGAB^, EGFP-Mena^ΔFAB^ and EGFP-Mena^ΔCC^ were taken under conditions identical to EGFP-Mena wild-type control.

### Biochemistry and pharmacology

To obtain total brain and body wall musculature homogenates, wandering instar larvae were filleted in dissection buffer (PBS, 1 mM EGTA, 1× *Complete mini* protease inhibitor cocktail; Roche, Indianapolis, IN, USA), transferred to 25 ml of lysis buffer (PBS, 0.05% Tween, 1 mM EGTA, 1× *Complete mini* protease inhibitor cocktail), homogenized and boiled for 10 min in 1× SDS Sample buffer (Sigma, St Louis, MO, USA). To dissociate the CNS from the muscle tissue, wandering third instar larvae were filleted in dissection buffer and the CNS was carefully removed from the body wall musculature. Ten brains and two muscle samples were homogenized in lysis buffer as described above. Samples were loaded into 10% SDS-PAGE gels (Lonza, Hopkinton, MA, USA), and analyzed by immunoblotting according to standard protocols. The following primary antibodies were used for western blotting: mouse anti-Ena 5G2 (DSHB), 1:20; rabbit anti-tubulin (Abcam, Cambridge, MA, USA), 1:50,000; and mouse anti-GFP (Living Colors Monoclonal Antibody JL-8; Clontech, Mountain View, CA, USA), 1:50,000. HRP-conjugated anti-rabbit and anti-mouse secondary antibodies (Jackson ImmunoResearch, West Grove, PA, USA) were used at a 1:10,000 dilution.

Latrunculin A (Sigma, St Louis, MO, USA) was dissolved in DMSO to a concentration of 1 mM to create a stock. The latrunculin A stock solution was then diluted to a concentration of 0.01 μg/μl in Ca^2+^-free saline. Wandering third instar larvae were dissected in Ca^2+^-free saline and then incubated for 10 min at room temperature in either Ca^2+^-free saline DMSO or Ca^2+^-free saline with latrunculin A. They were then fixed for 30 min in 4% paraformaldehyde and stained for anti-GFP as described above.

### Electron microscopy

Wandering third instar larvae were dissected in Ca^2+^-free saline. Their gut and internal organs were removed. Larvae were fixed in 2.5% paraformaldehyde, 5.0% glutaraldehyde and 0.06% picric acid in 0.1 M cacodylate buffer overnight at 4°C, and rinsed three times for 20 min in 0.1 M cacodylate buffer on ice. Brain and other debris were removed and the A1-A3 muscle area was cut out for further processing. The samples were then post-fixed with 1% osmium tetroxide and 1.5% potassium ferrocyonide in 0.1 M cacodylate buffer for 1 h on ice, rinsed three times for 5 min in deionized water, washed in maleate buffer twice for 10 min, incubated in 1% uranyl acetate in maleate buffer for 1 h, and dehydrated in ethanol series (50%, 70%, 95%, 100% and 100%) for 10 min each. Samples were then rinsed in propylene oxide for 20 min twice, then incubated in 1:1 propylene oxide and TAAB resin solution overnight. They were embedded in fresh resin at 65°C until hard. Sections were cut parallel to the surface of the muscle. Once a A2 6/7 muscle bouton was identified ∼50-90 nm sections were taken for a total of 5 μm. Sections were mounted on single slot grids, stained with lead and uranyl acetate, and imaged on a JEOL 1200EX – 80 kV electron microscope at 6500× and 25,000× magnification.

### Electrophysiology

The standard third instar larval body-wall muscle preparation developed by Jan and Jan (1976) was used for electrophysiological recordings (Zhang et al., 1998; Bao et al., 2005). Wandering third instar larvae were submerged in ice-cold HL-3 saline (Stewart et al., 1994), incised with a sharp razor blade along the dorsal midline and pinned out on a magnet dish with six pairs of metal pins. After removal of internal organs and fat tissues, the remaining body-wall muscle, along with the central nervous system (brain and ventral nerve cord), was rinsed three times with cold-HL-3 saline, and bathed in room temperature HL-3 solution. The nerve roots were cut loose with a pair of sharp scissors near the exit site of the ventral nerve cord so that the motor nerve could be later picked up by a suction electrode. The concentration of calcium contained in the HL-3 solution was 0.8 mM. The input resistance of each muscle was monitored, and those with values of 5 MΩ or higher were retained for final data analysis. The input resistance of the recording microelectrode (backfilled with 3 M KCl) ranged from 20 to 25 MΩ. Muscle synaptic potentials were recorded using an Axon Clamp 2B amplifier (Axon Instruments) and acquired by a Dell PC computer equipped with pClamp software. Following motor nerve stimulation with a suction electrode (100 µs, 5 v), evoked excitatory junction potentials (EJPs) were recorded. Three to five EJPs evoked by low frequency of stimulation (0.1 Hz) were averaged. For mini recordings, TTX (1 µm) was added to prevent unwanted evoked release (Zhang et al., 1998). The Mini Analysis program (Synaptosoft) was used to measure the amplitude of individual miniature EJPs (mEJPs or minis). At least 50 mini (up to 100) events from each muscle were analyzed to obtain the average amplitude of minis. Minis with a slow rise and falling time arising from neighboring electrically coupled muscle cells were excluded from analysis (Gho, 1994; Zhang et al., 1998). Quantal content was determined by the ratio of the average amplitude of EJPs and the average amplitude of minis. Analysis was completed using unpaired Student's *t*-tests with the Origin software. In addition, the Kolmogorov-Smirnov test was administrated when comparing mini sizes between preparations using Mini Analysis Program. The final figures were prepared using GraphPad Prism and Photoshop (Adobe).

## Supplementary Material

Supplementary Material
