# Supplementary material for: miR-8 controls synapse structure by repression of the actin regulator Enabled
Source: Development. 2014 May;141(9):1864–74. doi: 10.1242/dev.105791 (PMC3994775; doi:10.1242/dev.105791)
Supplement: Supplementary Material [file supp_141_9_1864__index.html]

miR-8 controls synapse structure by repression of the actin regulator Enabled — Supplementary Material 

# miR-8 controls synapse structure by repression of the actin regulator Enabled

## DEV105791 Supplementary Material

**Files in this Data Supplement:**

- **Supplementary Material**
