# Supplementary material for: miR-8 controls synapse structure by repression of the actin regulator Enabled
Source: Development. 2014 May;141(9):1864–74. doi: 10.1242/dev.105791 (PMC3994775; doi:10.1242/dev.105791)
Supplement: Supplementary Material [file supp_141.9.1864_DEV105791.pdf]

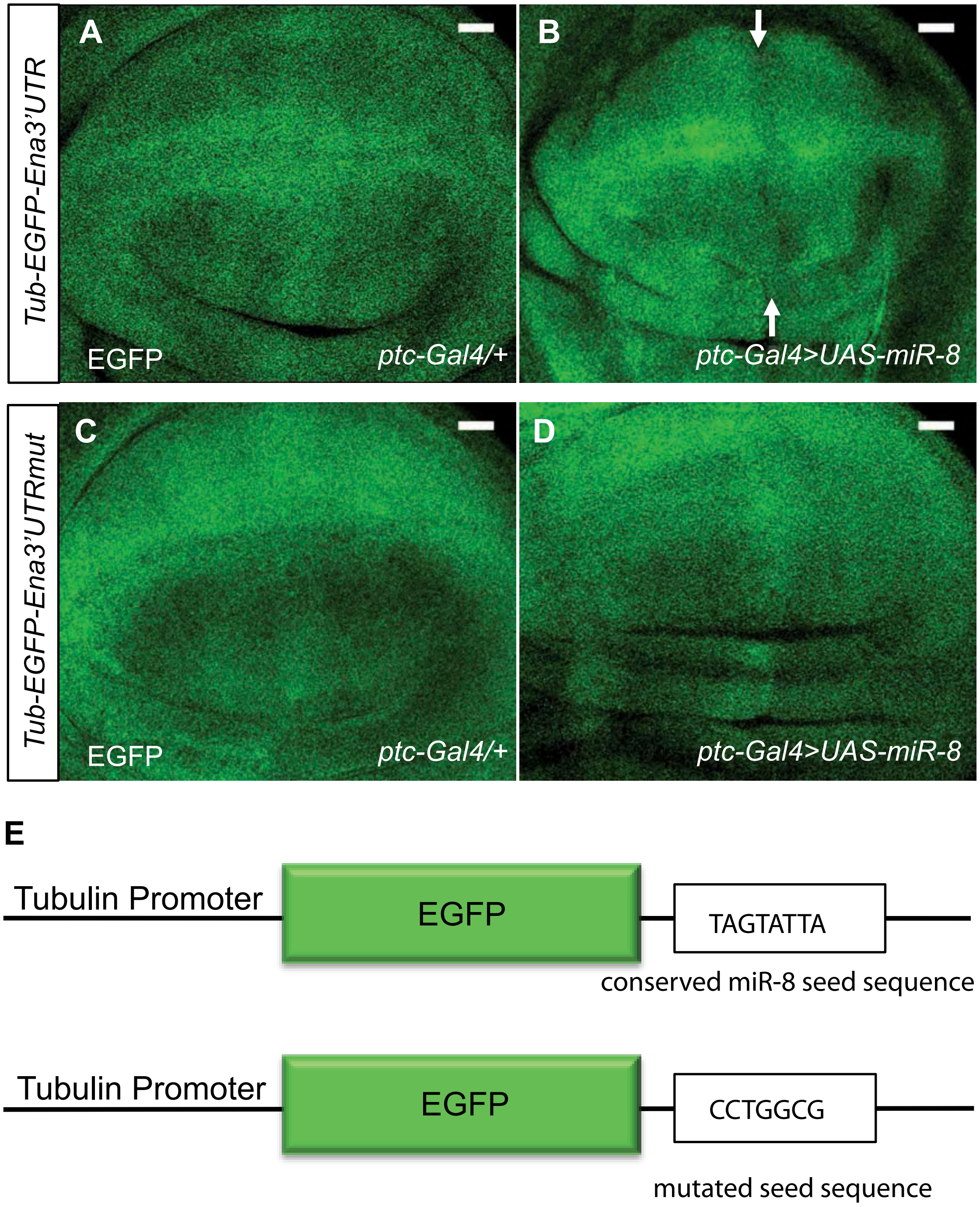

Supplemental Figure 1. Ena is an *in vivo* target of miR-8. (**A-D**) Images of third instar larval wing imaginal discs of *tub-EGFP-Ena3'UTR* and *tub-EGFP-Ena3'UTRmut* animals in a *ptc-Gal4* (**A, B**) or *ptc-Gal4>UAS-miR-8* expressing background (**C, D**), immunostained with an EGFP antibody. Cells expressing miR-8 along the anterior-posterior boundary of the wing pouch, indicated by white arrows display reduced levels of EGFP in **B**. Decrease in EGFP levels is not observed in **D**.

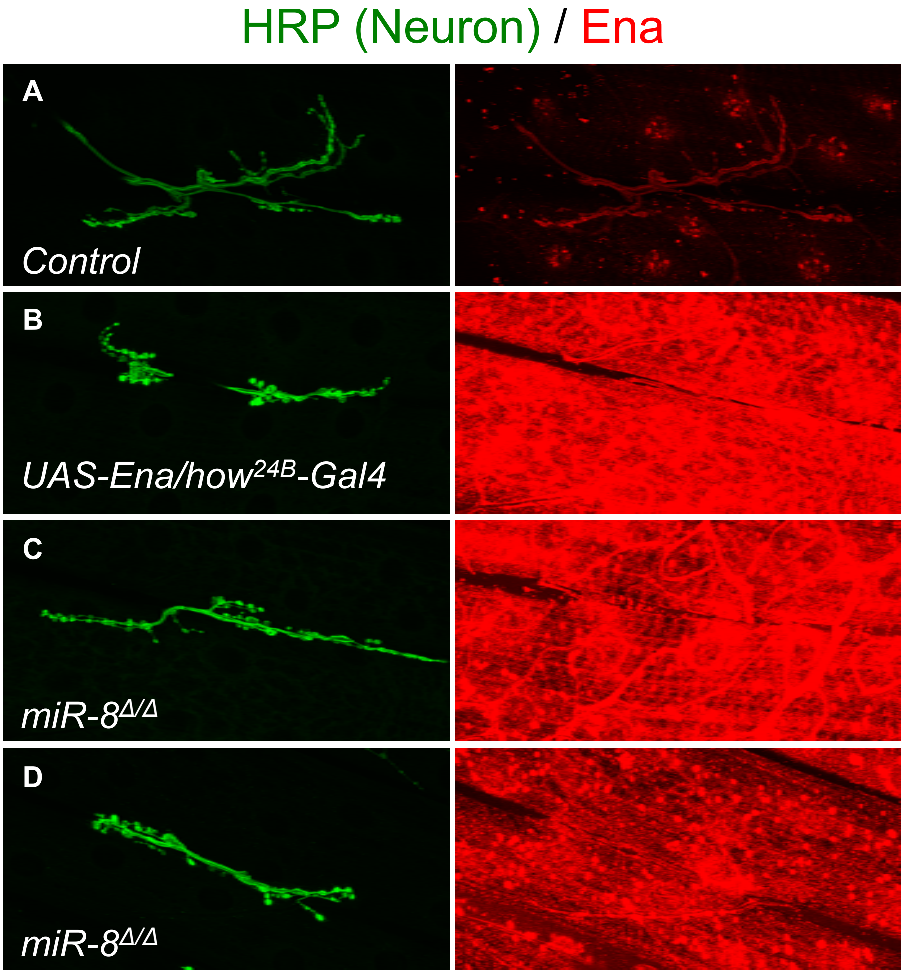

Supplemental Figure 2. Ena is highly over-expressed in muscle of miR-8 null animals. Images of *Drosophila* 6/7 NMJs immunostained with HRP (green) and Ena (red). Scale bar, 20μm. Ena expression in wild type control (**A**), over-expression of Ena with the pan-muscle *how*^{24B}*-Gal4* driver (**B**), and miR-8 loss (*miR-8*$^{\Delta/\Delta}$, **C-D**).
